# Immediate Postoperative Complications in Patients Undergoing CABG; Investigating the Role of Prior Coronary Stenting

**DOI:** 10.15171/jcvtr.2014.017

**Published:** 2014-12-30

**Authors:** Sohrab Negargar, Shahriar Anvari, Kyomars Abbasi, Elgar Enamzadeh

**Affiliations:** ^1^Cardiovascular Research Center, Tabriz University of Medical Sciences, Tabriz, Iran; ^2^Tehran Heart Center, Tehran University of Medical Sciences, Tehran, Iran

**Keywords:** Percutaneous Coronary, Intervention, Coronary Artery Bypass Graft, Outcome

## Abstract

***Introduction:*** Approximately 15 to 30% of patients undergoing percutaneous coronary intervention (PCI) will require repeated revascularization. There is an ongoing debate concerning the impact of prior PCI on subsequent coronary artery bypass graft (CABG) surgery. This study sought to compare immediate post-CABG complications between patients with and without previous coronary stenting.

***Methods:*** A total of 556 CABG candidates including 73 patients with previous coronary stenting and 483 patients without prior stenting were enrolled in this retrospective-prospective study. Demographic information, cardiac markers (CK-MB, Troponin T), and postoperative data including inotrope administration, intra-aortic balloon pump (IABP) use, bleeding, pathological electrocardiography (ECG) changes, and overall complications were compared between the two groups.

***Results:*** The mean age of the patients in stented group was significantly higher than that in unstented group (63.49±7.71 vs. 61.37±9.80 years, p=0.05). The mean serum level of Troponin T 12 h postoperation was significantly higher in the same group (323.26±33.16 vs. 243.30±11.52 ng/dL; p=0.03). Comparing the stented and unstented groups, the rates of inotrope use (17.8% vs. 7.2%; p=0.003), significant bleeding (15.1% vs. 4.3%; p=0.001), and overall complications (32.9% vs. 11.6%; odds ratio: 3.74 with 95% confidence interval of 2.13-6.55, p<0.001) were significantly higher in the former group. The association between overall complications and prior stenting was independent (odd ratio: 3.06). No significant connections were found between postoperative complications and stent number or type.

***Conclusion:*** A positive history of previous coronary stenting significantly increases the risk of immediate post-CABG complications.

## Introduction

Coronary artery disease (CAD) is one of the most common causes of death all over the world. Percutaneous coronary intervention (PCI) and coronary artery bypass graft (CABG) surgery are two principal recourses for patients with CAD.^1,2^

Evolving from straightforward balloon angioplasty of a single coronary stenosis to sophisticated multivessel stenting with employment of drug-eluting stents (DES), cutting-edge instruments, and advanced techniques,^3^ PCI is now ubiquitous and performed more commonly than in the past.^4^ In the Western countries the ratio of PCI to CABG is currently estimated to be over four to one.^5^

Despite this enthusiastic inclination toward PCI, it is well known that in comparison with CABG, PCI is along with higher rates of failure.^6^ In a study by Abbott et al^7^ the one-year rate of target-vessel revascularization was 5% for DES and over 9% for bare-metal stents.

This high rate of PCI shortcoming, and at the same time, with the number of stented patients on the rise, frequent encounters with CABG candidates with prior PCI(s) in the current practice is not a surprise anymore.^8,9^

Influences of preceding PCI(s) on CABG outcomes, however, have been not investigated adequately,^10^ and available reports in the literature are sometime contentious.^11-15^

This study sought to examine the effect of prior coronary artery stenting(s) on immediate postoperative outcome of CABG surgery.

## Materials and methods

### Study design and patients

After being approved by the ethics committee of a local university, medical records of 556 patients who underwent isolated CABG surgery at Tehran Heart Center from 2012 through 2013 were reviewed in this retrospective-prospective study. All patients were followed up during hospital stay.

The exclusion criteria were as follows: significant stenosis (>50% of the internal diameter) of the left main coronary artery; prior CABG; acute myocardial infarction within 24 h before CABG; concomitant cardiac/non-cardiac procedures; preoperative left ventricular ejection fraction (LVEF)<30%.

The patients were categorized in two groups; with (n=73) and without (n=483) prior intracoronary stenting.

### Study variables

Patients’ demographic information, previous diabetes mellitus, preoperative LVEF, postoperative serum levels of CK-MB and Troponin T 6 and 12 h after CABG, postoperative inotrope administration, postoperative intra-aortic balloon pump (IABP) use, postoperative bleeding requiring intervention, postoperative electrocardiography (ECG) pathological changes, and overall complications were documented and compared between the two groups of patients with and without intracoronary stent(s).

### Statistical analyses

Data analysis was performed using SPSS software (version 16.0). Normal distribution of data was checked using kolmogorov-smirnov test. Independent samples *t*-test, independent samples Mann-Whitney U test, Chi-square test, and Fisher’s exact test were used where appropriate. Logistic regression analysis was used for determining independent associations between variables (multivariate model). A p-value≤0.05 was considered statistically significant.

## Results

Demographics, preoperative variables, and immediate postoperative data in the two study groups are set out and compared in Table 1.

**Table 1:** Study variables compared between the two groups of patients with and without a history of previous intracoronary stent(s)

**Variable**	**Prior stent +** **(n=73)**	**Prior stent-** **(n=483)**	**p-value**	**OR**	**95% CI**
Age (year)	63.49±7.71 [49-80]	61.37±9.80 [30-48]	0.05^*^	-	-
Sex					
Male	53(72.6)	358(74.1)	0.78	1.08	0.62-1.88
Female	20(27.4)	125(25.9)			
Preoperative LVEF (%)	45.16±8.72 [25-60]	45.19±8.94 [15-60]	0.99	-	-
Diabetes mellitus	27(37)	179(37.1)	0.99	0.99	0.60-1.66
CK-MB					
6 h	28.99±2.94 [2.79-168]	24.91±0.92 [2.77-318]	0.19	-	-
12 h	23.26±5.89 [2.11-416.54]	19.63±2.35 [49-80]	0.58	-	-
Troponin T (ng/mL)					
6 h	419.41±41.65 [6.53-2555]	340.74±9.59 [4.20-1862]	0.07	-	-
12 h	323.26±33.16 [24.40-1492]	243.30±11.52 [4.40-3970]	0.03^*a^	-	-
Inotrope use	13(17.8)	35(7.2)	0.003^*^	2.77	1.39-5.54
IABP	3(4.1)	8(1.7)	0.17^b^	2.54	0.64-9.82
Significant bleeding	11(15.1)	21(4.3)	0.001*^b^	3.90	1.80-8.48
ECG change	3(4.1)	12(2.5)	0.43^b^	1.68	0.46-6.11
Any complication	24(32.9)	56(11.6)	<0.001^*^	3.74	2.13-6.55

Data are presented as mean±standard deviation [minimum-maximum] or frequency(%). CI: confidence interval, CK-MB: myocardial muscle creatine kinase, ECG: electrocardiogram, IABP: Intraaortic balloon pump, LVEF: left ventricular ejection fraction, OR: odds ratio ^a^Mann-Whitney U test ^b^Fisher’s exact test *p-value≤0.05 is significant.

The mean age of the patients in the group with prior stent(s) was significantly higher than the age of patients without prior intracoronary stent implantation (63.49±7.71 vs. 61.37±9.80 years, independent sample *t*-test p=0.05).

The two groups were comparable for sex (Chi-square test p=0.78), mean preoperative LVEF (independent samples *t*-test p=0.99), history of diabetes mellitus (Chi-square test p=0.99), men serum levels of CK-MB 6 h and 12 h postoperation (independent samples *t*-test p=0.19 and 0.58, respectively), mean serum level of Troponin T 6 h postoperation (independent sample *t*-test p=0.07), postoperative IABP (Fisher’s exact test p=0.17), and postoperative pathological ECG change(s) (Fisher’s exact test p=0.43).

The mean serum level of Troponin T 12 h postoperation was significantly higher in stented than in unstented patients (323.26±33.16 vs. 243.30±11.52 ng/mL, independent samples Mann-Whitney U test p=0.003).

Comparing the two groups, the rates of postoperative inotrope administration (17.8% vs. 7.2%, Chi-square test p=0.003, odds ratio: 2.77), significant bleeding (15.1% vs. 4.3%, Fisher’s exact test p=0.001, odds ratio: 3.90), and overall complication (32.9% vs. 11.6%, Chi-square test p<0.001, odds ratio: 3.74) were significantly higher in stented than in unstented group (Table 1).

In multivariate study analysis (logistic regression), the differences for age [p=0.05, Exp (B)=1.03], serum level of T Troponin 12 h postoperation [p=0.001, Exp(B)=1.00], postoperative bleeding [p=0. 01, Exp(B)=3.10], and overall complications [p<0.001, Exp(B)=3.06] remained statistically significant. The postoperative inotrope use was not an independent different factor in this regard (p=0.42).

Factors related to the stented group are summarized and compared between the stented patients with and without immediate postoperative complications in Table 2.

**Table 2:** Study variables relating to the stented group of patients stratified by the presence or absence of immediate postoperative complications

**Variable**	**Complication +** **(n=24)**	**Complication-** **(n=49)**	**p-value**	**OR**	**95% CI**
Age (year)	63.21±8.30	63.63±7.50	0.83	-	-
Sex					
Male	19(79.2)	34(69.4)	0.38	0.60	0.19-1.90
Female	5(20.8)	15(30.6)			
Preoperative LVEF (%)	46.13±10.57	44.69±7.73	0.56	-	-
Diabetes mellitus	6(25)	21(42.9)	0.14	0.44	0.15-1.31
Stenting to CABG (year)	7.90±2.78	7.15±4.04	0.38	-	-
Stent type					
DES	3(12.5)	14(28.6)	0.12		
BMS	12(50)	26(53.1)			
Unknown	9(37.5)	9(18.4)			
Stented artery					
LAD	15(62.5)	25(51)	0.36	1.60	0.59-4.34
LCX	5(20.8)	7(14.3)	0.51^a^	1.40	0.44-5.62
OM	1(4.2)	5(10.2)	0.66^a^	0.38	0.04-4.47
RCA	11(45.8)	29(59.2)	0.28	0.54	0.22-1.56
Stent number					
1	17(70.8)	31(63.3)	0.52^b^	0.71	0.25-2.04
2	6(25)	15(30.6)			
3	0(0)	2(4.1)			
4	1(4.2)	0(0)			
5	0(0)	1(2)			

Data are presented as mean±standard deviation or frequency(%). BMS: bare-metal stent, CABG: coronary artery bypass graft, CI: confidence interval, DES: drug-eluting stent, ECG: electrocardiogram, LAD: left anterior descending, LCX: left circumflex , LVEF: left ventricular ejection fraction, OM: obtuse marginal , OR: odds ratio, RCA: right Coronary Artery ^a^Fisher’s exact test ^a^ 1 versus ≥2 stents *p-value≤0.05 is significant.

Accordingly, no significant difference was documented as to the mean age of patents (independent samples *t*-test p=0.83), sex (Chi-square test p=0.38), mean preoperative LVEF (independent samples *t*-test p=0.56), the mean time between the first stenting and succeeding CABG (independent samples *t*-test p=0.38), stent type (Chi-square test p=0.12), the stented coronary artery including the left anterior descending artery (Chi-square test p=0.36), the left circumference artery (Fisher’s exact test p=0.51), the obtuse marginal branches (Fisher’s exact test p=0.66), and the right coronary artery (chi-square test p=0.28); and the number of stented coronary arteries (Chi-square test p=0.52).

## Discussion

To guide therapeutic strategies in candidates for CABG it is crucial to determine the real impact of prior PCI on subsequent CABG.

In the present work, first in the country to the best of the authors’ knowledge, the effect of prior intracoronary stenting on immediate postoperative outcome was examined in patients undergoing CABG.

Significantly older age, higher level of serum Troponin T 12 h postoperation, and higher rates of postoperative inotrope use, bleeding requiring intervention, and overall complications were connected to a history of previous stenting.

In a study by Pliam et al, in contrast to our findings, patients with history of stenting were significantly younger than patients without prior PCI.^16^ Similar finding was also reported in another study.^17^

This conflicting finding is possibly due to different characteristics of patients owing to peculiar guidelines picked up by cardiac surgeons and interventional cardiologists in dealing with patients with CAD.^18-21^

The adverse, independent role of older age in affecting PCI/CABG outcome in patients with CAD is well known.^22^

Incorporating an appropriately selected multivariate model, significantly associated complications with prior stenting, except for inotrope use, turned out to be age-independent. The independent odds ratio for development of overall postoperative complications in patients with stented coronary arteries was 3.06. In addition to age, this figure was independent of preoperative LVEF, another factor that plays substantial role in determining post surgical outcome in CABG patients.^16^

Such associations between previous PCI in patients undergoing subsequent CABG and worse postoperative outcome have been previously documented.^23-26^

In a study by Chocron et al^8^ on 2,489 candidates of CABG, the authors found that the risk of adverse CABG outcome was significantly higher in those with a positive history of prior PCI (hazard ratio: 1.53).

Massoudy et al^27^ studied 29,928 consecutive patients who underwent isolated first-time CABG and revealed that a history of previous PCI was significantly associated with in-hospital major adverse cardiac events (MACE, odds ratio: 1.5).

Patients with stent showed an increased risk of MACEs (hazard ratio: 2.784) than unstented patients in a study by Carnero-Alcazar et al^28^ on 1020 patients with CABG.

In another recent study on 7855 patients, Mannacio et al^29^ found that a history of previous PCI significantly increased postoperative MACE (odds ratio: 2.1).

In a study on 200 patients submitted for CABG surgery, Eifert et al^30^ found that administration of vasoactive inotropes and abnormally increased levels of troponin I were significantly documented more frequently in patients with prior PCI compared to those without previous PCI.

Our findings are in conformity with these reports. The odds ratio of association between previous intracoronary stenting and the occurrence of immediate postoperative complications in our work (3.06) was considerably higher than the reported values by the aforementioned studies (1.5-2.1).

There are multiple interactions between coronary arteries, coronary stents and the procedure of stent implantation. A cascade of inflammatory reactions initiated with every PCI procedure, coupled with an enhanced endothelial hyperplasia may lead to stent failure. The structural changes develop later after stent implantation may extend beyond the stented segment such as distal coronary section, which is the target area of a subsequent bypass graft anastomosis. Finally, endothelia dysfunction after stent implantation has been shown to be connected with decreased availability of vasculoprotective agents.^3,10,27,31-33^

In addition, when DES is employed deleterious interactions may occur between released active drugs and the arterial wall. These drugs may hinder endothelialization and at the same time, they may bolster tissue factor expression, resulting in a prothrombogenic environment. In addition, DES may be associated with exercise-induced paradoxical coronary vasoconstriction of the adjacent vessel segments.^10,34^

Last but not least, multiple and overlapping stents may occlude or obstruct coronary side-branches, leading to a compromised collateral flow and the development of focal infractions.^35^ It has been estimated that these focal infarctions may cause irreversible myocardial damage involving roughly 5% of the total left ventricular mass.^27^

We also investigated possible associations between parameters connected with prior stenting and immediate postoperative outcome in the stented group, separately. On the basis of these results, no significant associations were found between the overall complications and the interval time between stenting and CABG, stent type (i.e. DES vs. BMS), stented coronary artery, and the number of implanted stents.

Contrary to this, the postoperative course of multiply stented patients (>3) has been reported to be seriously compromised.^16^

This controversy stems possibly from small number of patients with over 2 stented vessels in the present work. Further studies with larger sample sizes may reveal more definite results in this regard.

This study bears some limitations that should be acknowledged here. Overall small sample size, and particularly small number of patients with multiple stents (>3) are major limitations of this study. Likewise, it was not clear whether the patients who received intracoronary stents were selected appropriately for PCI or not at the time.

In summary, this study showed that for stented patients the postoperative risk for in-hospital complications during subsequent CABG is escalated. Although this finding was also reported by some previous studies, which were mentioned earlier, a need for further studies is evident. To emphasize on this necessity, it should be reminded that the EuroSCORE scale, which is a risk assessment tool for cardiac surgery, has not yet incorporated the history of prior angioplasty as a prognostic factor.^36^

Although there are generally accepted guidelines for placing intracoronary stents, the matter of when to stop placing stents remains less understood.^37^

Finally, this classic stance that “in any patients with a history of previous PCI a successful subsequent CABG may be achieved” is critically under challenge now. It is pivotal to discriminate undoubtedly between patients who may or may not drive benefit from PCI.^10^ This strategy needs further studies.

## Conclusion

According to the findings of the present study, a positive history of previous coronary stenting significantly may increase the risk of immediate post-CABG complications.

## Ethical issues

The study was approval by the local ethics committee.

## Competing interests

Authors declare no conflict of interests in this study.
